# Cuffless blood pressure estimation based on haemodynamic principles: progress towards mobile healthcare

**DOI:** 10.7717/peerj.11479

**Published:** 2021-05-25

**Authors:** Takehiro Yamakoshi, Peter Rolfe, Ken-ichi Yamakoshi

**Affiliations:** 1 Institute of Liberal Arts and Science, Kanazawa University, Kanazawa, Ishikawa, Japan; 2 Division of Research & Development, MedicAlpha Corporation, Kobe, Hyogo, Japan; 3 Department of Automatic Measurement and Control, Harbin Institute of Technology, Nangang, Harbin, China; 4 Oxford BioHorizons Ltd., Maidstone, United Kingdom; 5 College of Science and Engineering, Kanazawa University, Kanazawa, Ishikawa, Japan; 6 Department of Orthopaedic Surgery, Showa University, Tokyo, Japan; 7 Division of Research and Development, Research Institute of Life Benefit, Nonprofit Organization (NPO), Sapporo, Hokkaido, Japan

**Keywords:** Blood pressure, Haemodynamic principle, Modified normalized pulse volume, Smartphone, Hypertension, Mobile health, Cuffless, Heart disease, Healthcare, Smart

## Abstract

**Background:**

Although cuff-sphygmomanometry is used worldwide in medical and healthcare fields, it is a fact that the use of an occlusive cuff to obtain blood pressure (*BP*) is troublesome and inconvenient. There have therefore been on-going efforts to devise methods that do not require the use of a cuff, almost all being based on the measurement of pulse wave velocity or pulse transit time, but so far few significant developments have been made, especially regarding measurement accuracy. We have previously reported a smartphone-based cuffless method using a linear multiple regression calibration model comprising of *BP* obtained with a cuff-sphygmomanometer as an objective variable and modified normalized pulse volume (*mNPV*: a measure of vasoconstrictive activity in a finger) and pulse rate (*PR*) as explanatory variables. This requires a number of subjects to construct a calibration model and thus is largely dependent on the accuracy due to the model. To address these drawbacks, we report here a new cuffless method to surpass considerably the results of our previous study as well as earlier works.

**Methods:**

With this method we can estimate *BP*, with much higher accuracy, using *mNPV* and *PR*, both also obtained from a smartphone-derived photoplethysmogram. The subject firstly performs a cuff-based *BP* measurement in parallel with the acquisition of *mNPV* and *PR* from a smartphone. These parameters are set as initial values (*BP*_c0_, *mNPV*_0_ and *PR*_0_; initial calibration procedure). Then, the estimated *BP* (*BP*_e_) can be calculated from the relation: “*BP*_e_ = (*BP*_c0_·*PR*·*mNPV*)/(*PR*_0_·*mNPV*_0_)”, which is derived from the so-called haemodynamic *Ohm’s* law. To validate this method, preliminary experiments using 13 volunteers were carried out to compare results from the new method with those from the cuff-sphygmomanometry, used as a reference.

**Results:**

Altogether 299 paired data sets were analyzed: A good agreement was found between the cuff-based and the estimated *BP* values, with correlation coefficients of 0.968 for systolic *BP* (*SBP*), 0.934 for mean *BP* (*MBP*) and 0.844 for diastolic *BP* (*DBP*). *Bland-Altman* analyses for the *BP*_e_ (*SBP*_e_, *MBP*_e_, *DBP*_e_) and the *BP*_c_ (*SBP*_c_, *MBP*_c_, *DBP*_c_) values also supported these comparison results. Mean absolute differences between the *BP*_e_ and the *BP*_c_ values in total subjects were less than 5 mmHg. Fairly good tracking availability in terms of time series data of the *BP*_c_ against the corresponding *BP*_e_ values was also confirmed in each subject during the study periods (1–2 weeks for 12 subjects and about 4 months for one subject).

**Discussion:**

The present study reported the successful development of the new cuffless *BP* estimation method, given as the status of a trial stage of investigation. This method could easily be used with various smartphones, smart watches, and finger-based devices, and it appears to have significant potential as a convenient substitute for conventional cuff-sphygmomanometers as well as for practical application to mobile healthcare.

## Introduction

It is well known that high arterial blood pressure (*BP*) is associated with increased risk of disease and mortality (James et al., 2014; Poulter, Prabhakaran & Caulfield, 2015). However, because *BP* can fluctuate by a significant amount (Parati et al., 2013) both on a beat-by-beat basis and in the long-term, it is important that it is checked frequently, especially in the case of the hypertensive subject. Non-invasive methods for checking beat-by-beat or intermittently obtained *BP* are preferred, and several are currently available, such as variations of the well-known *Riva-Rocci* mercury sphygmomanometer, as well as volume oscillometry (Yamakoshi et al., 1982; Parati et al., 2013; Yamakoshi et al., 2014), volume compensation (Yamakoshi, Shimazu & Togawa, 1980; Matsumura et al., 2017) and tonometry (Hansen & Staber, 2006).

During the use of conventional sphygmomanometry, an occlusive cuff must be applied to a finger, wrist, or the upper arm, and cuff pressure must be controlled to determine *BP* values. Despite their widespread use almost every minute of every day by tens of millions of people across the globe, arm-cuff-based *BP* measurement technologies in particular are still often considered to be inconvenient and cumbersome due to their reliance on the inflatable cuff. There have therefore been on-going efforts over recent decades to devise methods that do not require the use of the cuff.

The phrase “cuffless *BP*” is now a generic term for methods that determine *BP* without the use of a cuff. These approaches seek to avoid the discomfort or inconvenience associated with cuff inflation/deflation and also to obviate the need for a means of cuff pressurisation, which, in addition, increases the cost and complexity of the technology as well as complicating the measurement process. Almost all cuffless *BP* estimation methods proposed to date have been based on the measurement of pulse wave velocity (*PWV*) or pulse transit time (*PTT*) using various relationships between cuff-based *BP* and *PWV* or *PTT* (Payne et al., 2006; Gesche et al., 2012; Mukkamala et al., 2015; Sharma et al., 2017; Ding et al., 2017; Nabeel, Jayaraj & Mohanasankar, 2017).

In contrast to this, our group recently developed a much simpler method for cuffless *BP* estimation (Matsumura et al., 2018) based on a linear multiple regression model. Briefly, in this method, pulse rate (*PR*) and a modified normalized pulse volume (*mNPV*) (Lee et al., 2013) are used as explanatory variables, both of which can be obtained from the smartphone-derived reflectance-mode photoplethysmogram (*PPG*) (Matsumura & Yamakoshi, 2013; Lee et al., 2013). A linear multiple regression (calibration) analysis was made using the objective variable (*BP*; systolic (*SBP*), mean (*MBP* and diastolic *BP* (*DBP*) in each) measured by a cuff-based sphygmomanometer and the explanatory variables (*PR* and *mNPV*), to construct a *BP* calibration (regression) equation using a number of subjects. Then, the estimated *BP* can be calculated by the calibration equation, using *PR* and *mNPV* variables acquired by a smartphone (Matsumura & Yamakoshi, 2013).

The *mNPV* can be calculated from the amplitude of the pulsatile component (*PPG*_AC_) superimposed on the *PPG* signal, divided by the mean DC component (*PPG*_DC_) (i.e., *mNPV* = *PPG*_AC_*/PPG*_DC_). This is an alternate variable of *NPV* obtained with a transmittance-mode *PPG*, which was found to be almost equivalent and was originally proposed as a valid measure to represent vascular tone or sympathetic arteriola constrictive activity in a finger (Sawada, Tanaka & Yamakoshi, 2001).

It is clear that the accuracy of the estimated *BP* values by this method must largely be dependent on the magnitude of the variance in the regression, or in other words, on the accuracy of the *BP* calibration equation employed. This, in turn, means that the number of subjects used to determine the regression equation could greatly influence the estimated accuracy and error.

In order to avoid such erroneous factors in the regression and error analysis, we devised a new, more straightforward, cuffless technique so as to firstly pre-set cuff-based *BP* together with the *PR* and *mNPV* variables as initial values within an individual person. That person can then obtain the estimated *SBP*, *MBP* and *DBP* values using only *PR* and *mNPV* variables. In this study, we also used a smartphone (iPhone) to obtain the *PR* and *mNPV* variables. With this broad background, the objective of the present study was to validate this new technique through preliminary experiments to compare results from the new method with those obtained from a cuff-based sphygmomanometer as a reference method.

## Materials & methods

### Principle of *BP* estimation

The principle of *BP* estimation in this study is based on the well-known haemodynamic *Ohm’s* law, to be given as:

(1) meanBP(MBP)=cardiacoutput(CO)×totalperipheralresistance(TPR)

with the proviso that central venous pressure can be considered to be negligibly small as compared to *MBP* (Guyton & Hall, 1996). It has been reported that under resting conditions *CO* and *TPR* are essentially correlated with *PR* and *mNPV*, respectively (Obrist et al., 1978; Chau et al., 1978; Light, 1981; Sherwood, Dolan & Light, 1990; Sawada, Tanaka & Yamakoshi, 2001; Protogerou et al., 2007), and thus taking into consideration these evidential findings, we can reasonably assume that *CO* is approximately proportional to *PR* (*CO* ≒ (constant *k*_1_) × *PR*) and *TPR* is also approximately expressed as *TPR* ≒ (constant *k*_2_) × *mNPV*. A caveat is that this applies to the case of a subject who performs *BP* measurement under resting conditions, except for the case where they are carrying out any kind of exercise. Then, by making substitutions in Eq. (1) for *CO* and *TPR* we have:

(2) $$MBP=(k_{1}\times PR)\times (k_{2}\times mNPV)$$

Now, the subject is firstly requested to measure his/her own *SBP/DBP* using a cuff-based sphygmomanometer in parallel with the use of their smartphone to acquire *PR* and *mNPV* variables, pre-setting these variables *SBP*_c0_*/DBP*_c0_, *PR*_0_ and *mNPV*_0_ as initial values. This is the initial calibration (or setting) procedure. The subscript ‘c’ indicates the *BP* values determined by the cuff sphygmomanometer. We can calculate *MBP*_c_ from the following formula: *MBP*_c_ *= DBP*_c_ *+ (SBP*_c_ − *DBP*_c_*)/3*. The following equation, therefore, holds, using initial values as,

(3) $$MBP_{c0}=(k_{1}\times PR_{0})\times (k_{2}\times mNPV_{0})$$

Diving Eq. (2) by Eq. (3), we get the following equation, with the constants now eliminated, as:

(4) $$MBP/MBP_{c0}=(PR/PR_{0})\times (mNPV/mNPV_{0})$$

In this equation, *MBP*_c0_ (as well as *SBP*_c0_ and *DBP*_c0_) is obtained through the use of the cuff sphygmomanometer and thus is different from the quantity of *MBP* (*SBP* and *DBP* as well), being considered and termed as an estimated *MBP* (*MBP*_e_: also termed as *SBP*_e_ and *DBP*_e_). Equation (4) is therefore rewritten as the following simple equation:

(5) $$MBP_{e}=MBP_{c0}\times (PR\times mNPV)/(PR_{0}\times mNPV_{0})$$

Similarly, tentatively assuming that the same relationship holds with respect to *SBP*_e_ and *DBP*_e_, we can obtain the following equations as:

(6) $$SBP_{e}=SBP_{c0}\times (PR\times mNPV)/(PR_{0}\times mNPV_{0})$$

(7) $$DBP_{e}=DBP_{c0}\times (PR\times mNPV)/(PR_{0}\times mNPV_{0})$$

If either *SBP*_e_ or *DBP*_e_ is obtained by Eqs. (6) or (7), either one can also be simply calculated together with *MBP*_e_ as follows:

(6’) $$SBP_{e}^{*}=3MBP_{e}-2DBP_{e}$$

(7’) $$DBP_{e}^{*}=(3MBP_{e}-SBP_{e})/2$$

These equations mean that *BP*_e_ (*SBP*_e_*/MBP*_e_*/DBP*_e_) values can be estimated when the initial calibration procedure is done to determine *BP*_c0_ (*SBP*_c0_*/MBP*_c0_*/DBP*_c0_) along with *PR*_0_ and *mNPV*_0_. In other words, considering that *mNPV* corresponds to peripheral resistance, *BP* is to be linearly related to “*rate resistance-index product*” (*PR × mNPV*).

### Participants and ethical statements

A total of 13 volunteers (six males and seven females) participated in this study. All were Japanese, 19 to 73 years old, living their normal daily life in Tokyo, Kobe, and Sapporo cities. A total of 4 of the 13 subjects (No. 8, No. 11, No. 12 and No. 13 in Table 1) have suffered from grade 1 hypertension and have been taking antihypertensive agents every day. The others, 19 to 53 years old, had no current cardiovascular disease and did not take any prescription medications, although 2 subjects (No. 1 and 2), who have been living a normal daily life, appeared to have hypotension.

**Table 1:** Summarised subject information and measured values. Summarised values of mean (and *S.D*.) brachial *BP* and *PR*, iPhone-*PR* and *mNPV* and estimated *BP* values, and mean absolute difference MAD values obtained throughout the measurements in each subject. See text for symbols and further explanation.

Sub. no.	Gender	Age	BMI [kg/m^2^]	Number of measurements (measurement periods)	Mean (*S.D.*) brachial *BP* [mmHg] and *PR* [bpm] values				Mean (*S.D.*) PR [bpm] and *mNPV* [a.u × 10^−2^.], and estimated *BP* [mmHg] values							Mean absolute difference MAD (*S.D.*) [mmHg]		
					*SBP*_C_ <initial value>	*MBP*_C_<initial value>	*DBP*_C_<initial value>	*PR*_C_	*PR* <initial value: *PR*_0_>	*PR/PR*_0_	*mNPV* <initial value: *mNPV*_0_>	*mNPV*/*mNPV*_0_	*SBP*_e_ (*SBP*_e_*)	*MBP*_e_	*DBP*_e_ (*DBP*_e_*)	MAD of *SBP*_c_ & *SBP*_e_(*SBP*_e_*)	MAD of *MBP*_C_ & *MBP*_e_	MAD of *DBP*_C_ & *DBP*_e_(*DBP*_e_*)
1	Female	19	22.7	19 (14 days)	110 (6.3) <113>	80 (3.9) <81>	66 (3.0) <65>	80.7 (7.87)	80.8 (8.83) <76.8>	1.05 (0.118)	5.87 (1.78) <4.16>	1.41 (0.428)	107 (6.0) (110 (6.2))	84 (4.7)	71 (4.0) (73 (4.1))	3.8 (2.3) (2.4 (1.6)	3.9 (1.6)	5.8 (1.6) (7.3 (2.2))
2	Female	21	22.6	19 (12 days)	104 (3.7) <111>	81 (4.1) <85>	69 (5.0) <72>	58.7 (5.63)	58.6 (4.59) <66.2>	0.88 (0.071)	3.52 (1.23) <4.04>	0.87 (0.306)	103 (5.2) (103 (5.2))	79 (4.0)	67 (3.4) (67 (3.4))	2.9 (1.9) (2.9 (1.9))	2.0 (1.9)	3.1 (2.2) (3.1 (2.2))
3	Male	38	23.2	20 (14 days)	138 (7.3) <116>	103 (5.1) <90>	86 (4.4) <77>	66.5 (5.64)	67.2 (6.28) <52>	1.29 (0.124)	2.28 (1.01) <1.47>	1.55 (0.688)	136 (8.3) (136 (8.3))	106 (6.4)	91 (5.5) (91 (5.5))	4.3 (2.0) (4.3 (2.0))	3.3 (2.6)	4.8 (3.0) (4.8 (3.0))
4	Female	39	19.4	20 (12 days)	111 (3.5) <112>	85 (3.4) <86.7>	72 (4.4) <74>	71.3 (6.17)	72.1 (4.64) <72.6>	0.99 (0.066)	3.78 (1.18) <3.80>	0.99 (0.311)	112 (5.5) (112 (5.5))	87 (4.3)	74 (3.6) (74 (3.6))	4.2 (2.9) (4.2 (2.9))	3.9 (2.0)	4.1 (3.0) (4.1 (3.0))
5	Female	45	20	20 (11 days)	103 (5.9) <95>	71 (4.4) <65.7>	56 (4.1) <51>	69.1 (5.73)	68.3 (5.36) <71.1>	0.96 (0.078)	2.45 (0.656) <3.76>	0.65 (0.174)	104 (5.7) (104 (5.8))	72 (4.0)	56 (3.1) (56 (3.1))	2.8 (1.8) (2.8 (1.9))	1.8 (1.3)	1.9 (1.6) (1.9 (1.6))
6	Male	46	21.9	18 (19 days)	124 (7.6) <125>	92 (5.1) <90>	76 (4.8) <72>	66.1 (4.67)	68.3 (2.90)	0.94 (0.041)	4.10 (1.02) <4.42>	0.93 (0.233)	120 (7.1) (120 (7.1))	86 (5.1)	69 (4.1) (69 (4.3))	3.7 (2.0) (3.6 (2.0)	5.6 (2.8)	7.1 (3.1) (7.1 (3.1))
7	Female	53	23.1	20 (12 days)	105 (6.4) <110>	78 (5.0) <82>	64 (5.3) <68>	55.9 (3.57)	56.3 (3.33) <49>	1.15 (0.074)	6.22 (1.76) <3.40>	1.82 (0.515)	105 (6.9) (105 (6.9)	78 (5.1)	65 (4.3) (65 (4.3))	3.6 (2.1) (3.6 (2.1))	2.4 (1.5)	3.1 (2.3) (3.1 (2.3))
8^HY^	Male	67	24.9	19 (10 days)	139 (7.6) <142>	98 (4.7) <99.3>	77 (4.4) <78>	70.4 (3.57)	70.1 (3.64) <65.7>	1.07 (0.057)	7.52 (2.00) <5.90>	1.27 ((0.339)	140 (10.1) (140 (10.1))	98 (7.1)	77 (5.5) (77 (5.5-))	4.7 (2.5) (4.7 (2.5))	3.2 (2.1)	3.7 (2.4) (3.7 (2.4))
9	Male	67	27.3	19 (5 days)	125 (10.2) <118>	91 (6.2) <88>	74 (5.0) <73>	64.7 (5.10)	63.8 (3.65) <62.3>	1.02 (0.060)	8.48 (2.38) <9.16>	0.93 (0.259)	126 (10.2) (126 (10.2))	94 (7.6)	78 (6.3) (78 (6.3))	2.8 (1.7) (2.8 (1.7))	3.9 (2.4)	5.3 (3.3) (5.3 (3.3))
10	Female	71	20.6	17 (12 days)	121 (6.2) <117>	90 (4.6) <87>	75 (4.5) <72>	70.2 (4.25)	70.4 (4.77) <72.0>	0.98 (0.068)	7.51 (2.25) <5.45>	1.37 (0.413)	121 (7.3) (121 (7.3))	90 (5.4)	75 (4.5) (75 (4.5))	3.5 (2.7) (3.5 (2.8))	3.6 (2.1)	3.9 (2.4) (3.9 (2.4))
11^HY^	Male	72	24.9	61 (119 days)	138 (11.9) <134> <2^nd^: 128> <3^rd^: 128>	99 (8.8) <95.3> <2^nd^: 93.3> <3^rd^:90>	80 (8.0) <76> <2^nd^: 76> <3^rd^: 71>	64.1 (5.19)	64.1 (5.08) <68> <2^nd^: 72> <3^rd^: 67>	0.97 (0.084) 2^nd^ 0.84 (0.068) 3^rd^ 0.96 (0.071)	2.89 (1.35) <2.79> <2^nd^: 8.13> <3^rd^: 3.80>	0.69 (0.239) 2^nd^ 0.50 (0.260) 3^rd^ 0.75 (0.284)	138 (11.9) (138 (11.9))	98 (8.8)	78 (7.3) (78 (7.3))	2.8 (2.0) (2.8 (2.0))	3.7 (2.7)	5.0 (3.4) (5.0 (3.4)
12^HY^	Female	72	19.7	17 (6 days)	120 (9.3) <115>	86 (6.9) <84.3>	69 (6.4) <69>	68.1 (2.89)	69.7 (4.12) <77.8>	0.90 (0.055)	5.17 (1.91) <7.14>	0.73 (0.267)	118 (10.7) (117 (10.6))	86 (7.8)	71 (6.4) (70 (6.4))	4.1 (2.9) (4.1 (2.9))	2.3 (2.1)	2.8 (2.7) (2.7 (2.7))
13^HY^	Male	72	26.7	30 (14 days)	136 (13.4) <139>	97 (9.0) <98>	78 (7.5) <78>	65.1 (7.84)	65.0 (7.17) <65.9>	0.99 (0.11)	4.79 (1.76) <8.05>	0.60 (0.219)	136 (12.1) (136 (12.3))	99 (9.5)	81 (8.4) (80 (8.4))	3.3 (2.5) 3.1 (2.4)	3.7 (2.3)	5.1 (3.0) 5.1 (3.0)
															Total	3.5 (2.3) (3.3 (2.3))	3.4 (2.4)	4.4 (3.1) (4.5 (3.2)

**Notes:**

(a) superscript HY means hypertension with grade one, taking antihypertensive agents everyday.

(b) "Number of measurements" indicate the number excluded from the initial setting procedure in all subjects except that in the subject No. 11, in which the number is excluded from the initial setting and two times resetting procedures.

Written informed consent was obtained from all participants after we had provided them with a complete description of the study inclusive of measurement protocol and safety. This study was approved by the ethics committee of Showa University (Approval Reference Number: 2347) and conducted according to the principles expressed in the Declaration of Helsinki. This is not a replicated study.

### Apparatus and measurements

All participants had their own smartphone, including iPhone 7, 8, X, XS, 11 and 11 Pro (Apple Inc., Cupertino, U.S.A.). An experimental app (named “Exp_app”) was provided for this study, a modified version of the *iPhysioMeter* app (Matsumura & Yamakoshi, 2013) previously designed by us, and installed in each participant’s iPhone.

The Exp_app was essentially the same as the *iPhysioMeter* app, the details of which were reported elsewhere (Matsumura & Yamakoshi, 2013). Briefly, this application was rewritten to be compatible with iPhone 7 and later devices using iOS 13.3 or subsequent upgrades (Apple Inc., Cupertino, U.S.A.). The program allows the iPhone to serve as a reflectance-mode photoplethysmograph (*PPG*), producing the *PPG* signal by employing the CMOS camera and LED light built into the iPhone as a photodetector and light source, respectively. The *PPG* signal was acquired from the subject’s left index fingertip with a sampling rate of 60 frames per second.

Both *PR* and *mNPV* (*= PPG*_AC_*/PPG*_DC_) data were obtained from the *PPG* signal on a beat-by-beat basis via an auto-analysis algorithm included in the iPhone software. Any values that were significantly different from those over the prior 5 s time span were assumed to be outliers. A significant difference was defined as one that increased the standard deviation (*S.D*.) values associated with *PR* and *mNPV* by more than 8.0 beats per minute (bpm) or 0.25 arbitrary units (a.u.), respectively (Bland & Altman, 1986; Matsumura et al., 2014).

A brachial cuff-sphygmomanometer (DSK-1051, NISSEI, authenticated by the European Society of Hypertension (ESH), Milan, Italy; Japan Precision Instruments Inc., Hachioji, Japan) was provided for each participant. *BP* measurements were made by this device to produce the reference values of *SBP*_c_ *and DBP*_c_ as well as the calculated *MBP*_c_ (= *DBP*_c_ *+ (SBP*_c_ – *DBP*_c_*)/3*).

All of these data, including the raw *PPG* signals, were stored in the iPhone of each participant who e-mailed the data after each measurement to one of the experimenters to combine with all data for analysis.

### Measurement procedures

Experiments were carried out during normal daily life at each subject’s home, in a room temperature of around 24 to 26 °C, and humidity of around 45 to 55%. Throughout the measurements, the subject remained as motionless as possible, to sit for one min at rest in a chair with their left hand placed on a desk in front of them before starting the measurements; this was to reduce the appearance of movement artefacts. All of the subjects selected in this study knew very well how to operate the brachial cuff-sphygmomanometer provided, since they used their own sphygmomanometer routinely. It took about 40 s to obtain the brachial *BP* data and about 15 s to obtain the mean *PR* and *mNPV* values from averaging about 10 successive beat-by-beat data.

**Figure 1:**
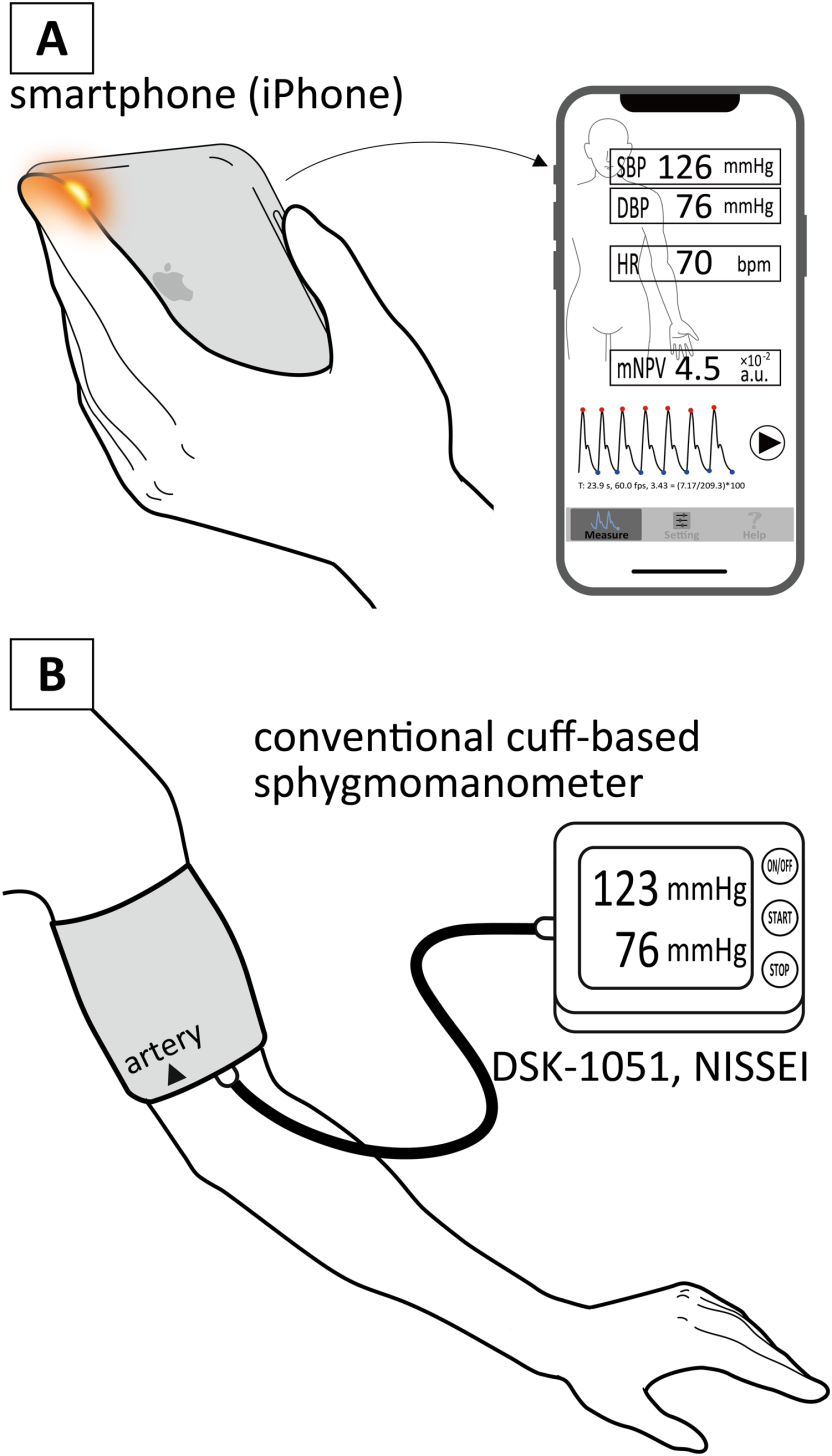
Illustrative drawings of measurement scenes. Illustrative drawings of measurement scenes by a smartphone (iPhone) (A) and a conventional cuff-based sphygmomanometer (B).

The participants were also asked to perform the *BP* measurements two or three times a day at any convenient time they wished. To make the contact condition between the iPhone CMOS camera and a subject’s fingertip almost the same, he/she was further reminded to place the iPhone softly on their left hand and keep their left index fingertip placed carefully over the camera, as shown in Fig. 1A. The fingertip was therefore just touching the camera. At this time, each subject was strongly recommended to mark their fingertip so as to place the camera almost in the exact same position for every measurement.

We have conducted preliminary experiments to measure simultaneously the contact pressure and *PPG* waveforms in this condition using a thin force sensor (FSR402, Interlink Electronics Inc., California, U.S.A.) fabricated into a donut shape. The pressure converted by the contact area was less than 2–3 mmHg, confirming that stable and reproducible *PPG* waveforms were obtained. Furthermore, the specially designed software (Exp_app) allowed the subject to watch their own *PPG* waveforms, as well as the calculated *PR* and *mNPV* values, on a beat-by-beat basis as displayed in the iPhone screen, in real time, during the measurement (also shown in Fig. 1A). If artifactual *PPG* waveforms, and/or unstable or outlying *PR* and *mNPV* values were observed, the subject stopped the procedure and then immediately restarted the measurement so as to minimize collection of erroneous data. This was also considerably helpful to reduce the appearance of movement artefacts and/or unstable *PPG* signals and for the subject to make a decision whether or not the signals were appropriate and/or acceptable.

After each *BP* measurement was made, the measurement by the iPhone continued to be carried out within about 30 s before major *BP* fluctuations occur, and then the subject manually input the obtained *BP* and pulse rate (*PR*) values (*SBP*_c_, *DBP*_c_ and *PR*_c_) to the iPhone. Each subject was requested to carry out the first *BP* measurement twice or three times to ensure that they could obtain almost the same *BP* values, within about 5 mmHg. The mean *BP* values (*SBP*_c0_*/DBP*_c0_) and the iPhone measurement values (*PR*_0_*/mNPV*_0_) were set as initial values (*i.e.*, this is the initial calibration procedure), being used for the calculation of *BP* estimation thereafter. The initial calibration procedure was made only once unless there was a considerable difference (over 30 mmHg or more) between the cuff-based and the estimated *BP* values.

The overall study periods lasted for 1–2 weeks to anticipate collecting about 20 paired data sets in each subject, but they could stop the process at any time according to their circumstances. One subject (No. 11 in Table 1) of the 13 volunteers proposed doing prolonged measurements at their convenience for about 4 months and 61 data sets were subsequently obtained from this subject.

### Data analysis

All of the data sent by e-mail were checked by observing the raw *PPG* signals as well as the obtained values of *SBP*_c_, *DBP*_c_, *PR*_c_, *PR* and *mNPV* in each subject. This was just to determine whether or not there were artefactual data and outliers, according to the criteria of standard deviation (*S.D*.) values of *PR* and *mNPV*. Actually, since the subject could watch their own *PPG* waveforms as well as calculated *PR* and *mNPV* values on a beat-by-beat basis displayed in the iPhone screen, as mentioned above, the data transmitted by e-mail from each subject were all essentially artifact-free and valid during the measurement period of 10 successive beat-by-beat data. The averaged *PR* and *mNPV* values were used for analyses. The initial setting values from the relevant subject (No. 1–10, No. 12 and No. 13) were also excluded from the data analyses. In subject No. 11 the initial setting and the second and the third resetting values were excluded. Altogether 299 paired data sets were finally obtained from the subjects.

Correlation analyses were made in each and in all of the subjects between the estimated *BP* (*BP*_e_) values (*SBP*_e_, *MBP*_e_ and *DBP*_e_) and the brachial *BP* (*BP*_c_) values (*SBP*_c_, *MBP*_c_ and *DBP*_c_) as the reference. *Bland–Altman* error analyses (Bland & Altman, 1986) for the *BP*_e_ and the *BP*_*c*_ values were also done in all of the subjects. Mean absolute difference (MAD) was further calculated for the evaluation of agreement between the *BP*_e_ and the *BP*_c_ values. A trend chart of the *BP*_e_ and the *BP*_c_ values obtained from one subject (No. 11 in Table 1) is presented to show *BP* tracking performance of this method for a longer period of time.

### Data availability

The data that support the findings of this study are available in *PeerJ*.

## Results

### Regression analyses in each subject

A total of 299 paired data sets were acquired for analyses obtained from 13 volunteer subjects. Table 1 summarizes the mean and *S.D*. values for all variables based on data acquired throughout the measurement process in each subject, along with the values of mean absolute difference (MAD [mmHg]) between the brachial *BP* (*SBP*_c_*/MBP*_c_*/DBP*_c_) and the estimated *BP* data (*SBP*_e_*/MBP*_e_*/DBP*_e_). *SBP*_e_* and *DBP*_e_* data calculated by Eqs. (6’) and (7’) are also included in Table 1 for reference.

### Agreement between estimated and cuff-based BP data

**Figure 2:**
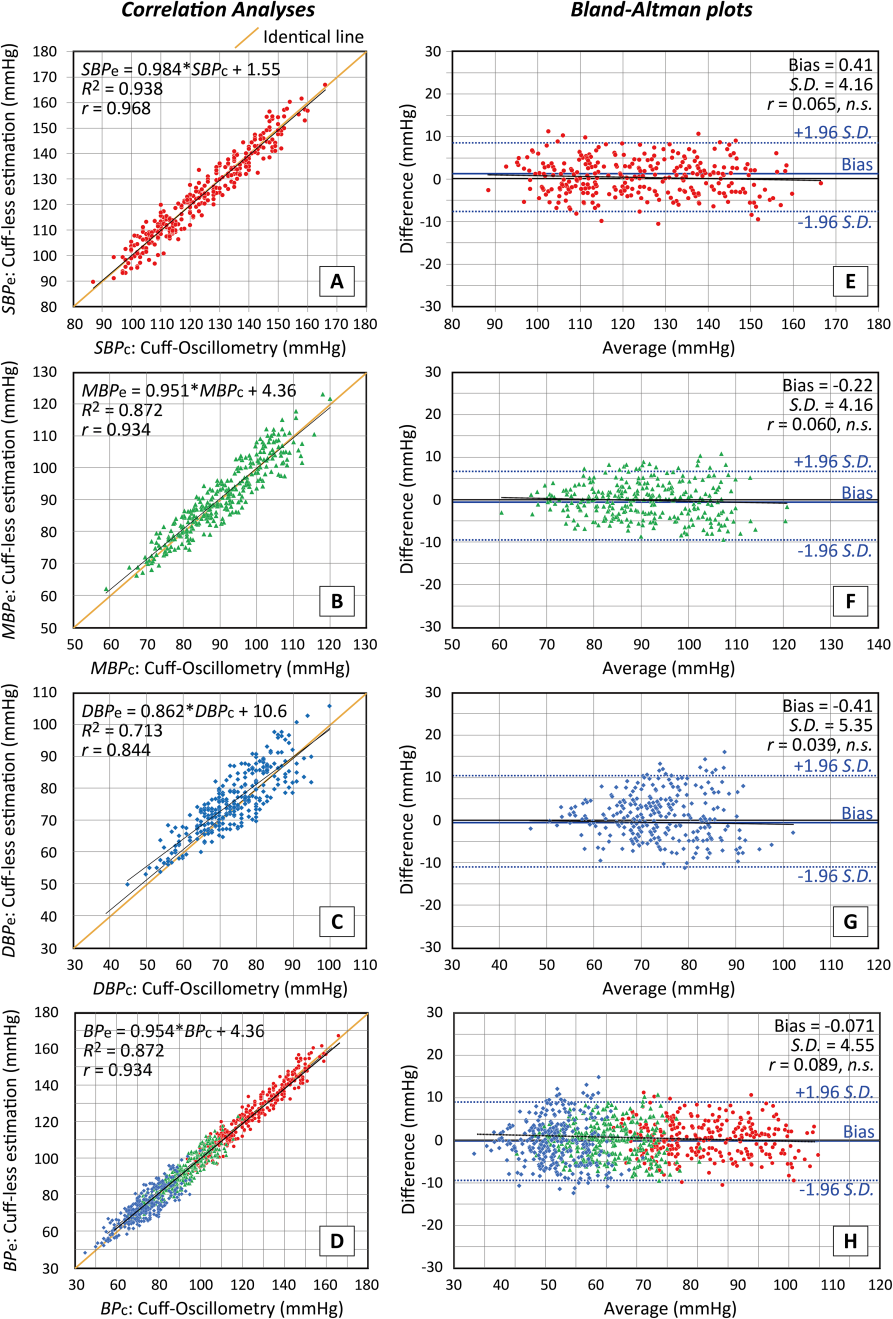
Accuracy of estimated values obtained with the proposed method. Accuracy of estimated values obtained with the proposed method. Scatterplots of *SBP* (A), *MAP* (B), *DBP*(C) and total *BP* (including all of S*BP*, *MBP* and *DBP*) values (D) estimated using a smartphone against the values determined with a brachial cuff sphygmomanometer (*n* = 299 for *SBP*, *MBP* and *DBP* and *n* = 897 for total). Lines of identity (solid orange lines), regression lines (solid black lines) and regression formula together with the coefficient of determination *R*^2^ and the correlation coefficient *r* values are shown in each scatterplot. Corresponding *Bland–Altman* plots of *SBP* (E), *MAP* (F), *DBP* (G) and total *BP* values (H). Solid and dashed lines represent the fixed bias and the limits of agreement (bias ± 1.96 *S.D*.). (*S.D*.: standard deviation), respectively. Average = (brachial + estimate)/2, Difference = brachial − estimate. *Pearson’s r* values are also presented in each graph.

Figure 2 shows scatter plots in all subjects based on paired *SBP*_e_, *MBP*_e_ and *DBP*_e_ data calculated by Eqs. (5)–(7) using the *PR* and *mNPV* values acquired from the iPhone against corresponding brachial data (*SBP*_c_, *MBP*_c_ and *DBP*_c_) determined by the cuff-based sphygmomanometer, along with the associated *Bland–Altman* plots. Such scatterplots using *SBP*_e_* and *DBP*_e_* were omitted since almost the same result as in Fig. 2 was obtained.

Figures 2A–2D are the scatter plots respectively for *SBP*_e_ *vs SBP*_c_, *MBP*_e_ *vs MBP*_c_, *DBP*_e_ *vs DBP*_c_ and total *BP*_e_ *vs BP*_c_, in which lines of identity (solid orange lines), regression lines (solid black lines) and regression formula together with the coefficient of determination *R*^2^ and the correlation coefficient *r* values are indicated. While Figs. 2E–2H are the *Bland-Altman* graphs in a similar manner to the above, in which fixed bias (M; drawn with solid lines) and *S.D*. values (limits of agreement of M ± 1.96 *S.D*.; drawn with dotted lines) together with *Pearson’s r* values, are presented.

MAD values between *SBP*_c_ and *SBP*_e_ (*SBP*_e_*), *MBP*_c_ and *MBP*_e_, and *DBP*_c_ and *DBP*_e_ (*DBP*_e_*) were respectively 3.5 ± 2.35 (3.3 ± 2.32), 3.4 ± 2.41 and 4.4 ± 3.09 (4.5 ± 3.18).

### BP tracking availability

**Figure 3:**
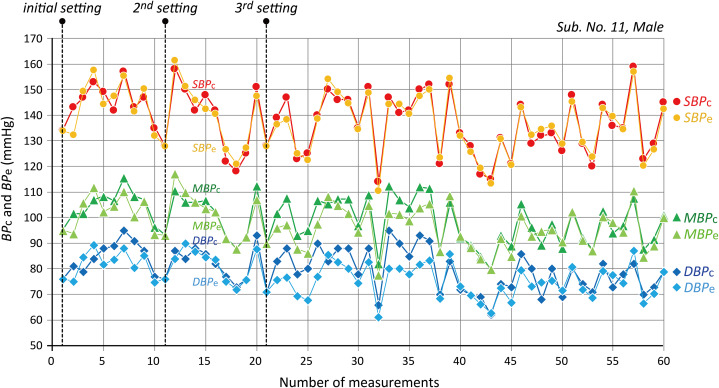
An example of a trend chart. An example of a trend chart of 61 data sets of *SBP*_c_*/MBP*_c_*/DBP*_c_ and corresponding *SBP*_e_*/MBP*_e_*/DBP*_e_ values obtained in one subject (No. 11) with the prolonged measurement period for about 4 months. See text for further explanation.

Fairly good tracking availability in terms of time series data of the *BP*_c_ and *PR*_c_ values by a cuff-sphygmomanometer against the corresponding *BP*_e_ and *PR* values by an iPhone, was confirmed in each subject within 1–2 weeks of a study period. Figure 3 is an example of a trend chart of 61 data sets of *SBP*_c_*/MBP*_c_*/DBP*_c_ and corresponding *SBP*_e_*/MBP*_e_*/DBP*_e_ values obtained in one subject (No. 11) with a prolonged study period of about 4 months, showing good *BP* tracking availability.

## Discussion

The goal of this work was to demonstrate a simple, suitably accurate, reliable for estimating *BP* without the use of an occlusive cuff. Although the present study constituted the status of a trial stage of development using a smartphone towards this goal, it is worth noting that estimated *BP* values by the new method described herein showed a good agreement with the measured values obtained by a cuff-based sphygmomanometer as a reference, provided that the subjects kept still, at rest, at home without any stressful tasks during the measurements. This means a limitation of this method at the present stage, thus indicating the need for a further study to be carried out under various measurement conditions. Therefore we discuss here advantages and disadvantages as well as technical improvements of this method, keeping such measurement limitation in mind.

It should firstly be noted that sequential measurements using the left hand were adopted as *BP* measurement by a brachial cuff-sphygmomanometer, followed immediately by a smartphone measurement, since subjects had to perform a series of measurement operations themselves which needed the use of their right hand. During the measurement, the subjects were requested to be at rest in a sitting position, so that any *BP* fluctuations could be considered to be acceptably small to enable the comparison analyses to be valid.

Taking also the real-life setting into consideration, in this study, all of the data were obtained from voluntary individuals during their normal daily life at home where they were exposed to little stress to cope by themselves, probably resulting in slight *BP* changes as compared to those in their normal away-from-home life. Actually, the *BP* variability in each subject was not so large, as inferred from the *S.D*. values shown in Table 1. It is also noted that the *BP* changes would be due mainly to the changes in the vascular-related variable *mNPV* rather than those in the cardiac-related variable *PR*, considering that the *S.D*. values of the ratio *mNPV/mNPV*_0_ were relatively large as compared to those of the ratio *PR/PR*_0_, as also shown in Table 1.

Under these measurement conditions during normal daily living, when plotted against *BP* values obtained using brachial sphygmomanometry as the reference (*SBP*_c_*/MBP*_c_*/DBP*_c_), *SBP*_e_, *MBP*_e_, and *DBP*_e_ data estimated from this method demonstrated very good correlation coefficients, *r* = 0.968 for *SBP*, *r* = 0.934 for *MBP* and *r* = 0.844 for *DBP* (see Fig. 2) with relation to neither gender nor to age. Also, MAD values between *BP*_*c*_ and *BP*_*e*_ data were acceptable within 5 mmHg. The good agreement between the two *BP* values strongly suggests that the *BP* calibration process conducted by each individual is very important; that is, each subject initially set their brachial *BP* data along with the *PR* and *mNPV* values (*BP*_c0_, *PR*_0_ and *mNPV*_0_) acquired from their own smartphone.

The availability of *BP* tracking was also found in all of the subjects except one (No. 11) without resetting this calibration procedure during the 1–2 weeks measurement period in the present study. In subject No. 11, who continued the study for a prolonged period of about 4 months, there was a considerable difference between *BP*_c_ and *BP*_e_ values of more than 30 mmHg; the reason for this is as yet unknown exactly, but perhaps is a displacement of the fingertip over the iPhone camera. This subject himself decided therefore to perform the calibration procedures twice instead of just once, as indicated by dashed lines in Fig. 3.

Overall, these results strongly suggest that this new cuffless technique, referred to as “*rate resistance-index product*”, based on the haemodynamic *Ohm’s* law, could allow smartphones, which are currently ubiquitous worldwide (Statista, 2016: Number of smartphone users worldwide from 2014 to 2020 (in billions)), to be employed as simple, stand-alone sphygmomanometers. Nevertheless, further research with larger in vivo and large-scale clinical experiments as well as technical improvements, as described below, would be required before this method could be fully accepted for routine clinical use.

As described in the Introduction, almost all cuffless *BP* estimation methods proposed previously have been based on the measurement of pulse wave velocity (*PWV*) or pulse transit time (*PTT*) with the use of various kinds of equations between cuff-based *BP* and *PWV* or *PTT*, on the basis of the *Moens-Korteweg* equation (Payne et al., 2006; Gesche et al., 2012; Mukkamala et al., 2015; Sharma et al., 2017; Ding et al., 2017; Nabeel, Jayaraj & Mohanasankar, 2017). A unique method combining *PWV* and *PPG* using machine learning to extract *PPG* features was recently reported (Kachuee et al., 2017). Also, smartphone-based *BP* estimation without cuff-based *BP* calibration using advanced machine learning algorithm was just recently reported (Luo et al., 2019). Apart from these two, all of these methods essentially require, sometimes frequently, a *BP* calibration that is based mainly on cuff-sphygmomanometry as a reference, and at present we cannot say for sure which of these methods is more convenient or reliable for routine use. One of the evaluation indices of these methods is measurement accuracy. The accuracies indicated by the *r* values in these previous methods were more or less 0.7 with a significant linear regression between cuff-based and estimated *BP* values, whilst in the present study the new method exhibited *r* values of 0.968, 0.934 and 0.844 respectively for the *SBP*, *MBP* and *DBP* data (Fig. 2). It should also be noted that the method described herein requires only a smartphone, so neither a *PPG* measuring unit nor a bio-amplifier for *ECG* detection are required. Taking these points into consideration, we believe that the present method is simple to use and has a reasonable accuracy as compared with the cuff-based sphygmomanometers with ESH standard used in this study: It is naturally noted that this method is available when the initial calibration is carried out using any type of commercial cuff-sphygmomanometer as a reference.

An important mathematical issue is that the assumption of constants *k*_1_ and *k*_2_ was used to derive the simplified relationships (Eqs. (5)–(7) expressed in the Methods section) based on the *BP* estimation in the present study, taking into account physiologically evidential findings from earlier literature (Obrist et al., 1978; Chau et al., 1978; Light, 1981; Sherwood, Dolan & Light, 1990; Sawada, Tanaka & Yamakoshi, 2001; Protogerou et al., 2007). Further research is, however, required to validate this issue through physiological experiments to make this method more secure and reliable. This validation aspect requires continuing investigation of the fundamental and mathematical background. However, our first essential step was planned simply to aim to compare the newly proposed method with cuff-based sphygmomanometry.

A further consideration, and possible disadvantage, with the use of the constants *k*_1_ and *k*_2_ in this new method, is that the assumed values of *k*_1_ and *k*_2_ do not always hold throughout measurements under resting conditions made over extended time periods. There is no choice at present but to avoid this issue by conducting a calibration procedure using the cuff-based *BP* measurements. It is therefore preferable to carry out, for example, weekly or monthly regular calibration procedures, although this is surely not ideal. However, this is not a major problem for people who wish to assess *BP* variation and regularly use a cuff-sphygmomanometer, since they can check *BP* values using only a smartphone when they like. The calibration procedures would be further simplified if the process of transferring the *BP* values to the smartphone was carried out automatically via wireless communication with a cuff-sphygmomanometer having a Bluetooth connection.

As has been shown above, good results were obtained by using Eqs. (6) and (7) to estimate *SBP* and *DBP* values respectively. These data were derived under the assumption that the haemodynamic *Ohm’s* law was valid. However, strictly speaking, this law actually applies to the mean value, *i.e., MBP* value. It is therefore necessary to investigate more appropriate estimation methods, based on the haemodynamic principles, for *SBP* and *DBP* values and this could then achieve even greater accuracy.

Careful contact and placement of the fingertip to cover the CMOS camera built into the smartphone is needed for stable determination of *mNPV*, and in fact also very important for obtaining an accurate *BP* estimation. Small differences in the contact state and the placement of the fingertip on the camera can produce a slight difference of the *mNPV* value, resulting in an erroneous *BP* estimation. It might be helpful for more practical use to design a jig embedded with a thin pressure sensor for gently fixing the fingertip and preventing relative movements as well as for monitoring the contact pressure between the fingertip and the CMOS camera. Further work is also suggested to search for a more suitable site such as the palm of a hand, to acquire a more stable *PPG* signal.

Although technical improvements such as those suggested above are needed for practical use, this new method offers a straightforward, sufficiently accurate (*r* > 0.84 and MAD < 5 mmHg) means of assessing *SBP*_e_, *MBP*_e_ and *DBP*_e_ with only a smartphone. At present it requires a calibration process using cuff-sphygmomanometry as a reference, however, a hybrid method in conjunction with, for example, our previous method (Matsumura et al., 2018) to remove such calibration procedure using *PR*_0_ and *ARI*_0_ could be worth considering, and is now under preparation to report elsewhere.

This new method, proposed as “*rate resistance-index product*”, based on haemodynamic principles, could potentially allow various types and models of smartphones and smart watches as well as finger-based pulse oximetry devices, to be used for *BP* estimation. It appears to have significant potential as a convenient substitute for traditional cuff-based sphygmomanometry.

## Conclusions

The present study reported the successful development of a new cuffless blood pressure (*BP*) estimation method, experimental validation, and progress towards mobile healthcare. With this method we could estimate *BP* (*BP*_e_; systolic (*SBP*_e_), mean (*MBP*_e_) and diastolic *BP* (*DBP*_e_)), with much higher accuracy than achieved with earlier work, based on the so-called haemodynamic *Ohm’s* law, using pulse rate (*PR*) and a modified normalized pulse volume (*mNPV*; a measure of sympathetic arteriola constrictive activity in a finger), both also obtained from a smartphone-derived photoplethysmogram (*PPG*). Preliminary comparison experiments using 13 volunteers under resting conditions at home clearly demonstrated a good agreement between the cuff-based *BP* (*BP*_c_) and the *BP*_e_ values through scatter plots and *Bland-Altman* error analyses as well as mean absolute differences between these two values. Fairly good tracking availability in terms of time series data of the *BP*_c_ values against the corresponding *BP*_e_ values was also confirmed in each subject during the study periods.

These results strongly suggest that this new cuffless technique, referred to as “*rate resistance-index product*”, based on haemodynamic principles, could allow smartphones, which are currently ubiquitous worldwide, to be employed as simple, stand-alone sphygmomanometers. However, further research with larger in vivo and large-scale clinical experiments as well as methodological and technical improvements, as described in Discussion, would be required before this method could be fully accepted for routine clinical use.

Nevertheless, the new method presented herein could potentially allow various types and models of smartphones and smart watches as well as finger-based devices, to be used for *BP* estimation. It appears to have significant potential as a simplified and convenient substitute for traditional cuff-based sphygmomanometry as well as for practical application to mobile healthcare.

## Supplemental Information

10.7717/peerj.11479/supp-1 Supplemental Information 1 The raw data of this study. A total of 315 original data sets obtained from 13 volunteer subjects.

## Additional Information and Declarations

### Data Availability

The following information was supplied regarding data availability: The raw data are available in the Supplemental File.
